# The visual analog rating scale of health-related quality of life: an examination of end-digit preferences

**DOI:** 10.1186/1477-7525-3-71

**Published:** 2005-11-14

**Authors:** Amir Shmueli

**Affiliations:** 1 Department of Health Management, The Hebrew University, POB 12272 Jerusalem, Israel

**Keywords:** Visual Analog Scale, End-digit preference, Health-Related Quality of Life

## Abstract

**Background:**

The Visual Analog Scale (VAS) has been extensively used in the valuation of health-related quality of life (HRQL). The objective of this paper is to examine the measurement error (rounding) explanation for the higher prevalence of VAS scores ending with a zero, and to provide an alternative interpretation.

**Methods:**

The analysis is based on more than 4,500 reported VAS valuations of own HRQL, included in two Israeli health surveys (1993 and 2000). Bivariate and logistic regression analyses are used.

**Results:**

The results show that reporting VAS scores ending with a 0 (...-20, ..0,10,20.....) decreases and scores ending with a 5 (...-15,-5,5,15,25,...) and with any other integer (...-12, -11,...1,2,...,92,..99) increases as VAS scores depart from 50, particularly when increasing up to 100. This pattern remains after controlling for personal characteristics determining the level of VAS.

**Discussion:**

Rounding true HRQL to the nearest 10's or 5's cannot explain the specific pattern found. It is suggested that this pattern corresponds to a S-shaped value function, where individuals tend to evaluate their HRQL as "gains" or "losses" relative to a reference point evaluated at 50. This particular reference score originates from being a traditional "passing threshold" and the scale's midpoint. Several implications of this interpretation to the measurement of HRQL are discussed.

## Background

Because of its simplicity and practical applicability, the Visual Analog Scale (VAS) has been widely used to elicit individuals' health value functions, either through measuring preferences for specific health states [1,2] or through evaluating their own health-related quality of life (HRQL) [3-5]. Recently, several studies examined the theoretical foundation of the VAS in relation to Von-Neumann-Morgenstern utility theory, and explored certain measurement problems such as end of scale aversion and spacing-out bias [2,6,7].

The present study focuses on end-digit preferences of the VAS scores, used to evaluate own HRQL. End-digit preference in reporting is not new, it was detected in 1940 in reporting age, and was later detected in blood pressure measurement, birth weight recording, and estimated gestational age [8,9]. The relative concentration of reported VAS scores ending with a 0 has been interpreted, as was done in the just mentioned studies in other contexts, as measurement errors, where people "round" their valuations to the nearest 10, while true HRQL is a continuous variable. However, it is shown that the closer the score is to 100 (perfect health), the higher the relative frequency of scores ending with an integer other than 0. Consequently, a different interpretation of the results is based on the assumption that no rounding is used, and respondents deliberately choose scores ending with 0, 5 or other integer to accurately reflect their HRQL. That interpretation, which implies an underlying S-shaped relationship between the VAS and true HRQL, is discussed.

## Methods

### The survey data

The data used in this study comes from two full sit-down health surveys – conducted in 1993 and in 2000 – of the Israeli Jewish urban population aged 45–75. Stratified (by settlement size) samples were used to represent the population studied. The 1993 survey included 1,999 individuals, while the 2000 survey included 2,505 individuals (for more details see [10]). Preliminary analysis showed that similar results (see below) are obtained for both years. Consequently, the final analysis reported below included the pooled two-year sample.

### The measurement of HRQL by the VAS

In both surveys, HRQL was valued in the following way: A card with a vertical scale ranging from -100 to +100, with unit marks (1s) and numbers appearing every five scores (at 5s and 10s), was presented to the respondents. The respondents were told that zero signifies HRQL associated with death, and 100 – HRQL associated with perfect health (regardless of age). The interviewers added that negative values are possible, meaning HRQL worse than that associated with death. The respondents were asked to report verbally the number on the above scale, which represents their general HRQL during the previous month.

### The statistical analysis

Bivariate and multivariate logistic regression analyses were used to show that the probability of VAS scores ending with an integer other than 0 or 5 differs in different ranges of scores. One may argue that such a pattern originates from the different characteristics of the respondents who chose different score ranges rather than from the scale itself. For example, persons enjoying very high HRQL might tend to report scores not ending with 0 or 5 more than other individuals. To examine that argument, selected personal characteristics, which are likely to affect the reported VAS score, were controlled for. These characteristics included: economic status (a set of 4 dummy variables representing the five categories: excellent, very good, good, fair and poor), ethnic origin (a set of 3 dummy variables representing the four categories: Asia-Africa, Europe-America, Israel and post 1990 immigrants from the former USSR), years of education, gender and age.

## Results

**Figure 1:**
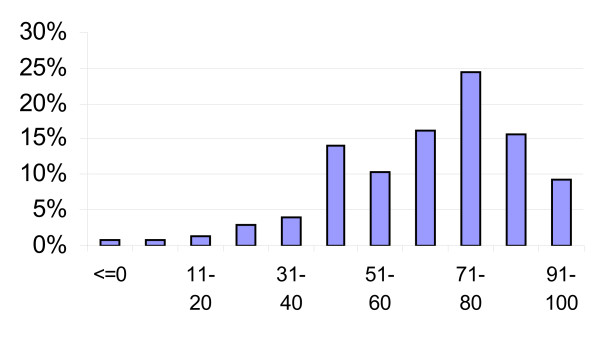
The distribution of VAS scores.

Figure 1 presents the distribution of VAS scores for the two years combined. The minimum score reported was -20. The mode of the distribution is 71–80, and the distribution is skewed to the left. For later reference, note the somewhat outstanding high frequency of the category 41–50.

**Figure 2:**
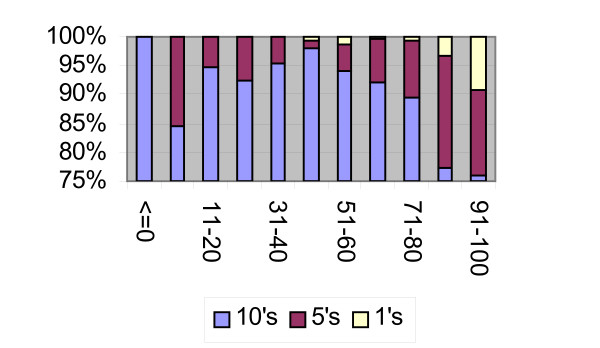
Proportions of scores ending with 0, 5, and other digits by valuation score.

Overall, 89% reported scores ending with 0 ("10s", -20, -10, 0, 10, ....,100), 9% reported scores ending with 5 ("5s", -15, -5, 5, 15,....95), and 2% reported a score ending with another integer ("1s" or all other scores, namely, -19, -18, ....1, 2, .....49, 51,....83, ...99). Figure 2 presents the (stacked) percentages of scores ending with 0, 5 and another integer by valuation categories, for the two years combined. The Figure shows that in the categories "< = 0" and "41–50", 98–100% of the scores are multiples of 10. In other words, all the scores below or equal to zero, are -20, -10 or 0. Similarly, 98% of the scores between (including) 41 and 50, equal, actually, 50.

Once the score is greater than 0 and lower than 40, or greater than 50, the percentages of 10s drop, and the proportions of scores ending with a 5 or another integer increase. For example, in the category "1–10", 85% of the scores equal 10, and 15% equal 5. This trend is more pronounced for scores greater than 50: in the 51–60 category, 94% of the scores equal 60, 4.5% equal 55, and 1.2% equal one of the remaining scores. In the upper category (91–100), 76% are equal to 100, 15% chose 95, and more than 9% are other scores in the category (92, 93, etc). The almost-steady increase in the proportions of scores not ending with a 0 or with a 5 is clear for scores higher than 40.

In order to test statistically the hypothesis that the proportion of scores ending with 0 is constant across the score-level categories (as is expected if just rounding was the issue), a logistic regression of the probability of a score ending with a 0 was run on the 11 score categories' dummy indicators. The Likelihood Ratio statistic, testing the hypothesis that all the score-level categories effects are equal, was 276.7 (DF = 10), which indicates that the hypothesis is rejected. Namely, the probabilities of a score ending with a 0 differ across the score-level categories. Controlling for the personal characteristics did not change the results. This means that the variable proportions of scores ending with a 0 and 5 (and hence of all other scores) by score range does originate from the VAS properties and not from the respondents' differing characteristics determining their score category.

**Figure 3:**
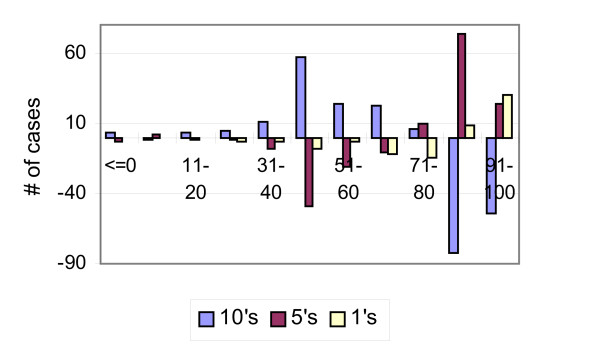
Actual minus expected (under a uniform distribution) number of cases ending with 0, 5, or other digit by valuation score.

Figure 3 shows the same results in terms of deviations of the actual number of cases from the expected ones, under a uniform distribution with rates equal the total's proportions of scores ending with a 0, 5 and other scores, by valuation score. The actual number of 10s is greater than the expected one in all scores up to score 80. It increases up to score range 41–50, and then drops. For score ranges 81–90 and 91–100, the actual number of 10s is smaller than the expected one. The deviations in the numbers of scores ending with a 5 by score range are almost an exact mirror image of the deviations of the number of 10s. They are negative and decreasing up to score of 50, and then negative and increasing up to 70, they continue to increase up to 90, and drop in the score range 91–100. The pattern of the deviations in the number of scores not ending with a 0 or 5 is similar to the one of the deviations in the number of scores ending with a 5, but smoother. Also, the deviations increase steadily from 71–80 up to 91–100.

## Discussion

Health-related quality of life (HRQL) is a latent continuous construct. VAS scores provide a measure of that unobservable variable. Much experience has shown that the VAS is easy to obtain, and respondents have no problems in scoring. The patterns presented above imply a particular relationship between the VAS reports and HRQL. The results, as shown in Figure 2 in particular, indicate a distinctive role for the score of 50. First, it is the score ending with a 0 with the largest concentration of responses. Second, disregarding for a moment negative scores, 98% of the individual scores within its neighboring score range are concentrated at its value, the highest concentration across all score ranges. Consequently, the percentage of scores ending with an integer other than 0, increases as the score range furthers away from 50, upward and downward. The score of 50 may be thus considered as an empirical reference or benchmark score (see below for an interpretation).

**Figure 4:**
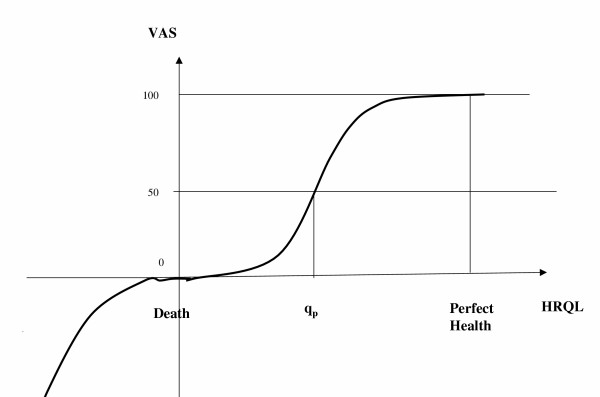
The implied shape of VAS scores as a function of true health-related quality of life.

Figure 4 presents the relationship between the VAS scores and true HRQL, which emerges from the above results. In Figure 4, q_p_, the true HRQL for which the VAS score is 50, is the true reference point of the scale. Levels of HRQL in the neighborhood of q_p _are not that different, leading respondents with HRQL in this range to round their VAS scores to 50. At that point, the curve is relatively vertical, since true HRQL is relatively constant. Scores in this range ending with integers other than 0 (say 41, 42,...., 49, 51,...59) indicate approximately the same level of HRQL – q_p_, so they are almost not reported. It takes 10 units of the scale (say 40, 60) to indicate a different level of HRQL.

The higher the VAS score (from 50 and up), the larger the difference in true HRQL (measured horizontally in figure 4) for a given difference in VAS (measured vertically). For VAS>91, each additional point on the score signifies relatively dramatically higher true HRQL. In this category every point is significant since HRQL is rapidly changing. For that reason, scores ending with an integer other than 0 are most frequent in this score range. Graphically, this translates into the curve being relatively flat (put inversely, the VAS is relatively constant over a relatively wide range of HRQL), and the curve is concave from below for HRQL values higher than q_p_.

A similar relationship between the VAS and HRQL holds for 0<HRQL< q_p_, with the curve being flatter for HRQL approaching that of death, so that the curve is convex from below for HRQL lower than q_p_.

A second threshold in the relationship is at HRQL = death, for which VAS = 0. As was argued above, the VAS for true HRQL worse than death is quite insensitive to the precise level of HRQL (scores ending with 0, the curve being graphically steep), and it takes differences of 10 points to indicate different levels of true HRQL. This threshold is defined, however, by the instructions. While the range of HRQL worse than death is extremely interesting and important, the analysis of this range is not very reliable, as only 10 persons (out of 4,504) reported negative scores on the VAS.

The value function in Figure 4 might represent valuation in a way similar to the one on which prospect theory is based [11]. Abstracting from uncertainty issues, prospect theory suggests that individuals do not evaluate states (e.g. levels of wealth) in their absolute value (as in Friedman-Savage utility theory) but as deviations (monetary gains or losses) relative to some reference point (e.g. the present level of wealth). Furthermore, the value function is concave (diminishing marginal value) for positive deviations (gains) and convex (increasing marginal value) for negative ones. Finally, the value function is steeper at each level of negative deviation than at the positive equal deviation. The value function depicted in Figure 4 following the empirical characteristics of the VAS reports, matches these characteristics. For non-negative HRQL, individuals evaluate their HRQL in relation to the reference value q_p_, which is the level of HRQL evaluated as 50. For HRQL better than q_p_, individuals consider the difference (HRQL-q_p_) as a "gain", and report a VAS value accordingly, with diminishing marginal value. For HRQL worse than q_p_, individuals consider the difference (HRQL-q_p_) as a "loss", and report a VAS value accordingly, with increasing marginal value. As is clear from Figure 2, the value function in Figure 4 is steeper for negative deviations (HRQL< q_p_) than for equal but positive deviations (HRQL> q_p_).

The distinctive role of the reference point q_p _evaluated by 50 is suggested by the data. Nevertheless, what can be the interpretation of these values? Two explanations can be offered. First, in Israel, as in many other education systems, the evaluation of the pupils' achievements is done by a grade on a 0–100 scale. On this scale, a grade of 50 is usually considered a "passing grade", where lower grades indicate a failure (in Israel, failing grades are commonly called "negative grades", reflecting the "loss" with respect to the passing grade 50 as a reference point recorded as 0). A second explanation sees 50 as simply the midpoint on the positive 0–100 scale. The psychometric importance of scales' midpoint is well known, e.g., the "midpoint bias", where (too) many respondents tend to choose the mid category from among an odd number of options.

The significance of q_p _evaluated as the mid-scale 50 is closely related to the "bisection procedure", where respondents matched, by a sequence of bisections, a number (magnitude) to brightness and loudness. This procedure was found to agree fairly closely with matching done by magnitude estimation (where numbers are directly matched to stimuli). Furthermore, for the magnitude estimation procedure, it is clearly stated that: " [....] stimuli should be presented in a different irregular order to each subject, but the first stimulus is usually chosen from among those in the *middle *region...." ([[12], p. 428], emphasis added).

## Conclusion

A critical assumption of all studies using VAS-derived valuations is that the VAS is a proper interval scale, namely, the passage from 2 to 4 (2 points), for example, bears the same cardinal meaning as the passage from 56 to 58, and from 98 to 100 (as with a thermometer), with 0 and 100 arbitrarily chosen as reference points. If that assumption holds true, the analysis in this paper showed that the VAS valuation scores represent a value function as depicted in figure 4, with actual reference point at q_p _(valued at 50), and not a straight line diagonal connecting 100 (HRQL of perfect health) and 0 (HRQL of death).

The implications for HRQL measurement are that the verbal valuation is done in a relative way, with regard to a reference level of HRQL valued at 50. The exact level of HRQL, which is valued as 50, is unknown, and may vary across individuals. If it does vary across individuals, the comparison of VAS scores between individuals is problematic, since though the 0 and 100 anchors are well defined, they are actually used by the respondents to define the effective reference point q_p _evaluated as 50. Naturally, it does not mean that the S-shaped VAS score over- or under-estimate true HRQL relative to the common interpretation of VAS, since true HRQL is unknown. It does exclude, however, the notion of a reference point being the mean score in the population. The end-digit properties of written VAS evaluations done with the aid of a marked ruler are expected to be similar.

A straightforward test of the argument advanced in this paper would be to examine the distribution of VAS evaluations of own HRQL with respect to scores ending with 0, 5 and other integer by score-ranges in other populations, in particular where the traditional educational achievement scales are based on other scales, e.g., the A, B, C,...F grading system.
